# Novel, low-cost bioreactor for *in vitro* electrical stimulation of cardiac cells

**DOI:** 10.3389/fbioe.2025.1531731

**Published:** 2025-02-03

**Authors:** Joseph P. Licata, Jonathan A. Gerstenhaber, Peter I. Lelkes

**Affiliations:** Department of Bioengineering, College of Engineering, Temple University, Philadelphia, PA, United States

**Keywords:** stem cells, organoids, computational modeling, 3D printing, cardiomyocyte, electric field modeling

## Abstract

**Introduction:**

The successful implantation of laboratory-grown cardiac tissue requires phenotypically mature cardiomyocytes capable of electrophysiological integration with native heart tissue. Pulsed electrical stimulation (ES) has been identified as a promising strategy for enhancing cardiomyocyte maturation. However, there are discrepancies in the literature as to best practices for promoting cardiac differentiation using ES.

**Methods:**

This study presents a novel, 3D printed bioreactor that delivers *in vitro* ES to human induced pluripotent stem cell-derived cardiomyocytes (hiPSC-CMs), promoting cell maturity and functional readiness for implantation. Finite element analysis and mathematical modeling were used to model the fluid dynamics and to characterize in detail the delivery of pulsatile electrical signals, providing precise control over stimulation parameters such as voltage, current, and charge.

**Results:**

The bioreactor developed here provides an easy-to-use, inexpensive platform for culturing hiPSC-CMs under the influence of ES and low-shear fluid flow for enhanced nutrient availability, while its “drop-in” design facilitates real-time observation of cultured cells. The electrical stimulation provided is controlled, modeled, and predictable, enabling reproducible experimental conditions and promoting comparability across future studies. Human induced pluripotent stem cell-derived cardiomyocytes (hiPSC-CMs) grown in the bioreactor with ES showed improved differentiation and an enhanced ability to respond to external electrical pacing signals.

**Discussion:**

By offering a standardized platform for ES-based cardiomyocyte maturation, this bioreactor aims to accelerate advancements in cardiac tissue engineering. Future research will explore how variations in ES parameters influence cardiomyocyte phenotype and maturation, contributing to a deeper understanding of cardiac cell development and optimization for therapeutic applications.

## 1 Introduction

Myocardial infarction is known to cause cell death after a prolonged period of impaired blood flow, leading to significant and lasting heart tissue damage (Ertl and Frantz, 2005). Current therapies to aid in the prevention of complications after myocardial infarction include drugs to lower cholesterol and blood pressure, lifestyle changes, stents for blocked arteries, implantable devices (such as a pacemaker or implantable defibrillator), or, in the most extreme cases, transplants of entire hearts (Aronow, 2009). However, these therapies do not directly repair damaged tissue in the heart. To that end, numerous attempts have been made at integrating stem cell-derived cardiomyocytes (CMs) directly into infarcted hearts (Silver et al., 2021), either as single-cell implantation (Lee et al., 2024) or as a lab-made cardiac patch (Liu et al., 2024). To date, significant challenges remain that prevent the success of these treatments, such as cell retention (Wu et al., 2021), risk of teratoma formation due to incomplete differentiation of stem cells into mature (Kawamura et al., 2016), or lack of electrophysiological integration (Gepstein et al., 2010; Liao et al., 2010). One step toward solving these issues will be the consistent generation of stem-cell derived, mature CMs that upon transplantation can electrically couple to the existing cardiac tissue via connexins (Roell et al., 2007) and respond to electrical signals to contribute to a controlled heartbeat (Mandel et al., 2012).

Electrical signals are important for the development of cardiac tissue *in vivo* (Thomas et al., 2018; Hirota et al., 1985). *In vitro* electrical stimulation (ES) has previously been explored as a regulator of cardiac cell maturation and function, particularly in human induced pluripotent stem cell-derived CMs (hiPSC-CMs) (Ronaldson-Bouchard et al., 2019; Ma et al., 2018; Hernández et al., 2018). The outcomes of these studies, however, have been inconsistent. While most studies have shown that some amount of directly coupled, pulsatile ES is beneficial for CM maturation, there is not yet a consensus on the best stimulation parameters to use, including the frequency, amplitude, and pulse duration of the stimulation signals (Dai et al., 2021). While the majority of the published studies has been conducted using an electric field strength in the range of 3–6 V/cm (Ruan et al., 2016; Crestani et al., 2020; Chan et al., 2013), other studies report ES at as low as 2 V/cm (Hirt et al., 2014) or as high as 9 V/cm (Ronaldson-Bouchard et al., 2018). Studies also vary significantly in both the frequency (Tandon et al., 2011) and duration of ES (Geng et al., 2018; Yoshida et al., 2019) signals, as well as the developmental timepoint (Crestani et al., 2020; LaBarge et al., 2019) for starting such stimulation. Individual studies may vary multiple parameters at once, for example: amplitude, pulse frequency, duration, and developmental timing of the electrical stimulation. Given that some of these studies (Gabetti et al., 2023; Hu et al., 2024) report the results of multiple changed parameters without proper controls, it is difficult to distinguish which parameters are of primary importance to directing cardiac differentiation.

Bioreactors are dynamic cell and tissue culture vessels used to provide stimuli to cells grown *in vitro*, allowing for the recapitulation of environmental cues not typically found in static culture conditions (Licata et al., 2023). Although recent bioreactors have been developed to deliver electrical signals to cardiac cells, the authors often failed to provide enough detail about these signals to ensure the work can be reproduced (Gabetti et al., 2023; Hu et al., 2024). In this study, we present a bioreactor designed for precise, controlled electrical stimulation of cells grown *in vitro* in 2D monolayers or as 3D spheroids This bioreactor is designed for low-shear fluid mixing for enhanced nutrient availability, while also allowing for easy live optical monitoring throughout the duration of an experiment using standard microscopy. The electrical stimulation characteristics of this bioreactor were modeled computationally and tested experimentally, allowing for precise control over the delivered electrical signals. Here, we show that hiPSC-CMs cultured in this bioreactor under the influence of controlled, characterized, reproducible electrical stimulation showed enhanced cardiac maturation/development compared to standard culture conditions.

## 2 Methods

### 2.1 Bioreactor design and fabrication

**FIGURE 1:**
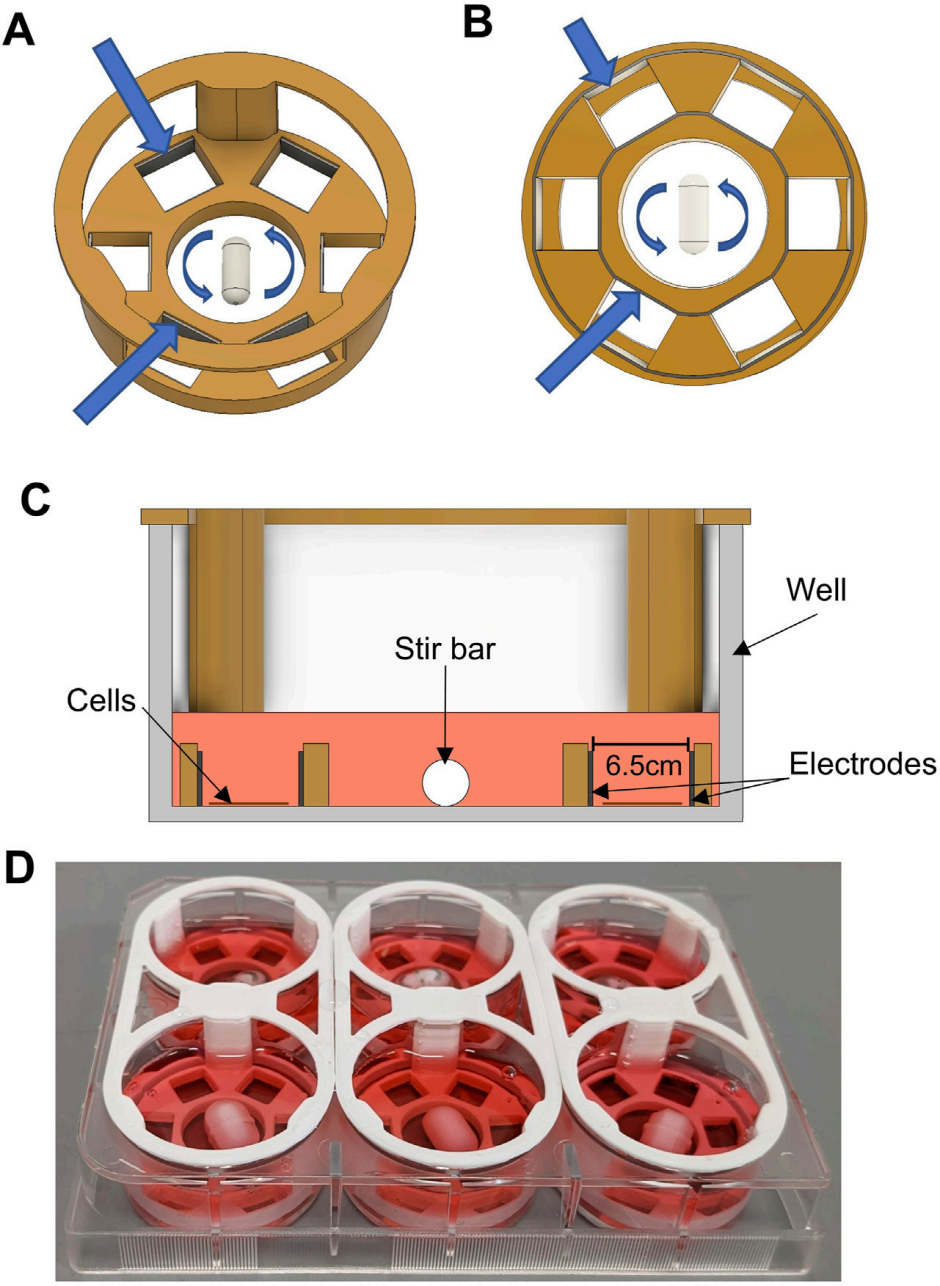
Bioreactor Design. **(A)** CAD rendering of the custom bioreactor design. 3D-printed plastic is shown in orange. The stir bar in the center is shown with arrows denoting rotation. Larger blue arrows point toward inner and outer electrodes. **(B)** Bottom view of bioreactor design. Arrows point to inner and outer electrodes, showing how they are each a single, continuous metallic strip that slots into grooves in the bottom of the design. **(C)** Cross-sectional view of bioreactor design, showing the device inside of a well of a six-well plate. Cell culture media is shown in pink. The area intended for cell culture is located between the parallel electrodes within two of the six sub-wells. **(D)** Photograph of six bioreactors assembled and placed into a six-well plate.

The bioreactor (Figure 1) is designed to “drop in” to a six-well plate and consists of a central mixing chamber surrounded by 6 electrode-lined “sub-wells” wherein the cells will be cultured. 3D printed parts for the bioreactor were designed using Fusion 360 (Autodesk Inc., San Francisco) and part files are available upon request. Parts were printed in Nylon12 on a Formlabs Fuse printer (Formlabs, Somerville MA). We recently verified the cytocompatibility of Nylon12 for use with hiPSCs (Licata et al., 2024). Stimulating electrodes were cut as strips of 316 stainless steel foil (McMaster Carr) and inserted into the bottom of the 3D-printed plastic part. After assembly, the bioreactors were washed with soap and warm water before being soaked overnight in distilled (DI) water to remove any remaining soap residue. Bioreactors were then sterilized via steam autoclave (Getinge, Gothenburg, Sweden), using a 15-minute exposure at 121°C with a 20-minute drying time, and allowed to cool before insertion into six-well polystyrene tissue culture plates.

### 2.2 Computational modeling

Computational modeling of the electric field and currents modeling were performed using the Ansys Electronics Desktop software package (Ansys, Inc., Canonsburg, PA), using models either built in the software or imported from Fusion 360. Modeling was performed using Maxwell 3D with a DC Conduction solution type, including multiphysics for insulator field calculations, similar to previously reported (Aragón et al., 2020).

Fluid dynamic modeling was performed using Ansys Fluent. 3D models of the bioreactor were either built in software or imported from Fusion 360. Computational modeling was performed using a k-omega (SST) Viscous model, using frame motion to simulate the rotation of a stir bar in the center of the device, similar to previously used methods to calculate fluid mixing in small bioreactors (Shafa et al., 2019).

### 2.3 Electrical characterization

An NI ELVIS II system (National Instruments, Austin, TX) with included software was used to measure voltage and current across a single bioreactor, submerged in cell culture media. Total bioreactor current was calculated by measuring the voltage before and after a small (1Ω) resistor in series with the bioreactor. Assuming the current in the bioreactor could be modeled as a simplified Randles cell (a circuit consisting of a capacitance and polarization resistance in parallel, and a solution resistance in series with that pair) (Nelms et al., 2003), the circuit element parameters were estimated by first measuring the current for a 500 m square pulse at varying voltages. Current measurements immediately after pulse initiation and after the current had reached a steady state were used to estimate the solution resistance and polarization resistance, respectively. The relationship between current and time was used to estimate capacitance, similar to previously described procedures (Lu et al., 2013). Charge injection was measured by calculating the total positive current during a 10 m pulse, and the total negative current following the end of said pulse, at varying voltage potentials. Custom MATLAB (Mathworks) software was developed to analyze all voltage and current data.

### 2.4 Stem cell maintenance culture

Human induced pluripotent stem cells (hiPSC; ATCC ACS-1026) were maintained on Corning Matrigel (Fisher Scientific)-coated T25 plates using mTeSR + media (STEMCELL Technologies, Vancouver, Canada). The media was changed every 2 days, and the cells passaged at a 1:15 split ratio according to the manufacturer’s specification when approximately 60%–70% confluent, using ReLeSR cell dissociation reagent (STEMCELL Technologies, Vancouver, Canada).

### 2.5 Directed cardiac differentiation

Differentiation of hiPSCs into cardiomyocytes was initiated according to an established protocol (Lian et al., 2012) with slight modifications. Cells were maintained as described above. Upon reaching 90% confluence, the maintenance media was changed to cardiac differentiation media (CDM) which consisted of RPMI 1640 (ThermoFisher Cat: 11875093) supplemented with 2% B-27 Minus Insulin (ThermoFisher, Cat: A1895601) and 200 μg/mL L-Ascorbic acid 2-phosphate (Sigma-Aldrich, A8960). On Day 0, maintenance media was changed for CDM supplemented with 2.5 µM CHIR99021 (Cayman Chemical, 13,122). On Day 1, this media was changed for fresh CDM without CHIR99021. On Day 3, media was changed to CDM supplemented with 2 µM of the Wnt-inhibitor XAV-939 (Tocris Bioscience, Bristol, UK). On Day 5, the media was changed to fresh CDM. On Day 7, the media was changed to Cardiac Differentiation Media with Insulin (CDMI), which is identical to CDM, except the B-27 Minus Insulin is replaced for B-27 Supplement containing insulin. From this point onward media was changed every 3 days.

### 2.6 Cardiac spheroid formation

On Day 10 of differentiation, the cells (estimated at >70% cardiomyocytes via flow cytometry) were dissociated using TrypLE™ Express (ThermoFisher Cat: 12604013). Cells were collected in CDMI and briefly centrifuged to exchange the spent media with fresh CDMI. After counting, cells were plated in a 96 well round bottom plate (Corning, CLS7007) at about 10,000 cells per well and centrifuged at 100 × g for 3 min to encourage aggregation at the bottom of the well. After ∼2 days, compact, beating spheroids could be observed.

### 2.7 Electrical stimulation

**FIGURE 2:**
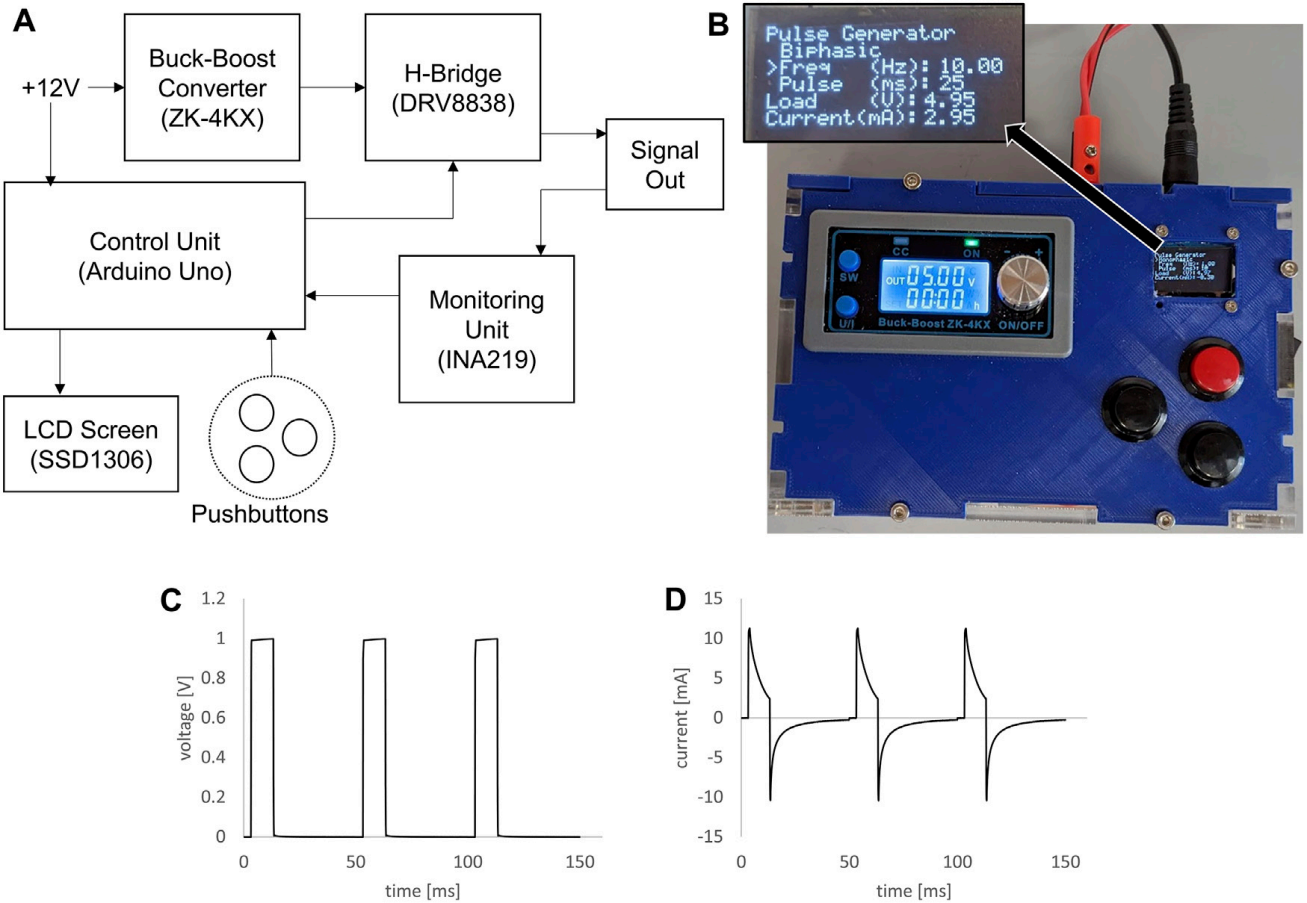
Custom signal generator for bioreactor. **(A)** Block diagram of electrical components for the custom signal generator. **(B)** Photograph of assembled signal generator in custom housing, inset showing zoomed view of the LCD screen. **(C)** Voltage plot of 1V, 10 ms, 20 Hz pulse train generated by the custom signal generator and applied to a bioreactor. **(D)** Plot of measured current through a bioreactor for the same signal parameters.

*In vitro e*lectrical stimulation (ES) of cardiomyocytes was performed in our bioreactor. Cells were seeded in the sub-wells of our custom bioreactor (see Figure 1) placed in six-well plates and differentiated according to the above protocol. For monolayer culture, stimulation began on Day 7 and concluded on Day 12. For spheroid culture, Day 12 spheroids were transferred into our bioreactors (housed in six-well plates) and exposed to ES for 5 days. For both culture types, stimulation parameters were as follows: 1 Hz stimulation, 10 m monophasic square pulses, 5 V/cm (3.25 V with an electrode separation of 6.5 mm). These parameters were chosen for this study as they are similar to those chosen in previous studies (Ma et al., 2018; Yoshida et al., 2019; Ahadian et al., 2017). Stimulation signals were generated by a custom-built stimulation hardware (see Figure 2A) controlled by an Arduino Uno (Arduino, Italy). A buck-boost converter (Amazon.com, ZK-4KX) was used as a variable power supply, a DRV8838 h-bridge for power switching (Pololu, Las Vegas, United States) and an INA219 (Adafruit, New York, United States of America) was used for monitoring current and voltage.

### 2.8 Optical analysis of cardiomyocyte beating

Videos of beating hiPSC-derived cardiomyocytes were recorded using a high-speed (240fps) cell phone camera (Pixel 6a, Google) connected to a Nikon Diaphot inverted phase contrast microscope equipped with an environmental control chamber set to 37°C. The captured video recordings were cropped and analyzed using ImageJ (NIH) with the open-source plugin MUSCLEMOTION (Sala et al., 2018). The extent and the speed of contraction profiles from MUSCLEMOTION were then further analyzed using a custom MATLAB script. Beating rates in at least 4-5 different areas from at least 3 biological replicates were analyzed and averaged for each condition.

### 2.9 Immunostaining and imaging

Cells grown on glass coverslips were washed with 1x PBS and fixed in 4% paraformaldehyde in PBS for 15 min at room temperature. Cells were permeabilized in 0.1% Triton X-100 (Sigma-Aldrich) for 10 min before blocking for 1 h at room temperature in 5% FBS, 5% goat serum, 0.01% Triton X-100 in PBS. All antibodies were diluted in antibody staining buffer: 2% FBS, 0.01% Triton X-100 in PBS. Mouse anti-sarcomeric alpha-actinin (EA-53 Sigma Aldrich) was diluted 1:500, and rabbit anti-connexin43 (C6219, Sigma-Aldrich) was diluted 1:2000. Primary antibodies were incubated simultaneously for 1h at room temperature. Cells were washed five times for 5 min each with PBS-T wash buffer (0.05% Triton X-100 in PBS) before secondary antibody incubation for 1 hr. Secondary antibodies used were FITC-conjugated goat anti-Mouse IgG (Thermofisher, 31,569) diluted 1:1,000 and Alexa Fluor 568 conjugated goat anti-Rabbit IgG (Thermofisher, A-11036) diluted 1:1,000. After incubation, the cells were washed 5 times for 5 min each in PBS-T before 10 min incubation with DAPI solution, 0.5 ug/mL in PBS. Cells were washed 2x with PBS before mounting on glass slides with Fluoromount G (Thermofisher, 00-4958-02). Slides were then imaged on an Olympus IX-81 using a 60x oil objective. Multiple images of each of at least 3 biological replicates were analyzed in ImageJ (NIH).

### 2.10 Statistics

All statistical analyses were performed using Microsoft Excel or JMP statistical software. A two-way ANOVA test was used to compare beating rates over time between both groups, and *post hoc* analysis was performed using Tukey’s Honest Significant Difference (HSD) test to identify specific differences. Statistical significance of morphological cell features was determined using a student’s T-test, with significance set at p < 0.05.

## 3 Results

### 3.1 Bioreactor design

A major goal of this study was to design, test, and implement an inexpensive bioreactor capable of both electrical stimulation and enhanced fluid flow, while continuously monitoring cell/organoid characteristics via fluorescence and phase-contrast microscopy. To this end, we developed the bioreactor depicted in Figure 1. Shown in Figure 1A is an angled overhead rendering of the main design of the bioreactor that consists of a large central mixing area, where a stir bar is spun using a magnetic stir plate to provide gentle media mixing, and six outer “sub-wells” where the cells are grown. The inner and outer walls of each sub-well are lined with stainless steel foil strips, to act as the electrodes that provide the electrical stimulation (ES). As seen in Figure 1B, the inner and outer electrodes are each a solid piece that is inserted into grooves in the bottom of the plastic part and are designed to be flush with the bottom of the part. The plastic body was designed to be amenable to biomanufacturing by 3D printing. For these studies the device was printed in Nylon12, a material that has previously been shown to be cytocompatible for use with naïve stem cells and cardiac differentiation protocols (Licata et al., 2024).

After assembly and sterilization, this entire device was “dropped” into a well of a six-well plate. The cross-sectional view (Figure 1C) shows where the cells were seeded and grown within each sub-well and between two parallel electrode surfaces. Because the cells were growing directly on the culture surface of the well plate, cells were easily observable throughout culture and experimentation. The device was designed to press-fit into the well, without any significant gaps between the plastic part and the bottom of the well. With about 2 mL of culture media added to each well, the media was able to gently flow over the wall of the 3D printed piece, allowing for effective mixing between the sub-wells and the central mixing area (see section below on computational modeling). A photograph of a set of assembled bioreactors in a six-well plate is shown in Figure 1D.

Electrical stimulation for the bioreactor was created by a custom electrical signal generator (Figure 2). The block diagram in Figure 2A shows how the electrical components were connected. An Arduino Uno running custom software was used as the control unit, changing the timing of the pulses generated according to user input and displaying information on a small LCD screen. The signal voltage was controlled using an off-the-shelf buck-boost module. Shown in Figure 2B is a photograph of the final device, contained in custom housing, with the signal output wires connected via banana jacks in the rear of the housing. This signal generator created clean voltage controlled square waves (Figure 2C), and the resulting current through a single bioreactor was then measured (Figure 2D).

### 3.2 Computational modeling

To determine the homogeneity of both fluid flow and electric field within the sub-wells of the bioreactor, computational modeling was performed using ANSYS modeling software. An ideal bioreactor design would show a homogenous electric field within the culture areas, to ensure that all cells experience the same electrical activity. In terms of fluid mixing, it has previously been demonstrated that fluid shear stress below 1 Pa can be considered “low shear” conditions (Varma and Voldman, 2015). An ideal amount of shear flow within the culture areas will enhance the availability of nutrients and oxygen to cells while keeping shear low enough to prevent any additional effect from exposure to high shear, such as off-target differentiation to endothelial cell types (Huang et al., 2021).

**FIGURE 3:**
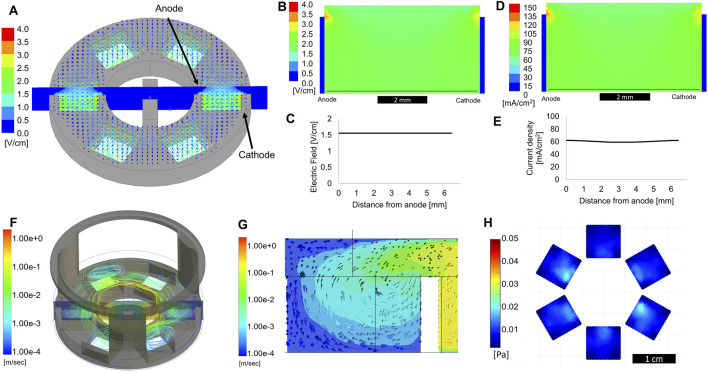
Computational modeling of electric field and fluid flow. **(A)** 3D electric field modeling of entire bioreactor. Cathode and anode are defined as labeled, with a 1 V applied potential used for modeling purposes. The cross-section view shown in the center is used for further 2D plots. **(B)** Cross section view of the electric field a single sub-well. The thin black line denotes the electric field trace 100 μm from the bottom of the well, plotted in **(C)** as electric field vs distance from the anode. **(D)** Cross-section view of current density within a single sub-well. The thin black line denotes the current density trace 100 μm from the bottom of the well, plotted in **(E)** as current density vs distance from the anode. **(F)** Computational modeling showing fluid flow streamlines originating from a rotating stir bar in the center of the device. **(G)** Cross section of the 3D model showing a side-view of a single sub-well. Arrows show the direction of fluid flow. **(H)** Shear stress at the bottom of the device, within the sub-wells, average of 5 s of modeling.

Figure 3A shows the electric field modeled for the entire bioreactor in 3D. The electric field was modeled using a 1 V potential applied from the inner anode to the outer cathode. A cross-sectional plot of the electric field can be seen across the center of the device, which was used for generating 2D plots for further analysis. The model used an electrical conductivity value of 1.7 Siemens/m for the media within the well, a previously determined value (Lang et al., 2015). Shown in Figure 3B is the electric field in a cross-section of a single sub-well. The electric field was essentially homogenous across the well, with some reduction in field strength toward the top of the well. This was due to the liquid extending above the edge of the electrodes. However, as shown in Figure 3C, the electric field was consistent across the width of the well in the area where the cells were cultured, approximately 100 μm from the bottom of the well. Figure 3D shows the electric current density plot in a cross-section of the same model. While the current density dropped slightly toward the center of the well, due to the spreading of current in the media about the tops of the electrode plates, this non-uniformity was minimal (Figure 3E). Though this modeling shows the homogeneity of ES throughout the bioreactor, it did not account for transient effects of pulsed ES and the effects of double-layer capacitance at the electrode-electrolyte interface, which will be discussed in the next section.

Computational fluid dynamic modeling showed how liquid flowed in the bioreactor while the stir bar was spinning at 100 RPM. During stirring, fluid flowed over the top of the plastic piece separating the mixing area and sub-wells, as seen by the streamlines and cross-section view in Figure 3F. While the fluid in the mixing area approached a velocity of 1 m/s, it was flowing slower than 1 mm/s when reaching the bottom of the sub-wells, where the cells were located (Figure 3G). Analysis of the flow direction indicated that fluid was circulating outward from the center mixing area, while media from the culture areas was circulating back into the center. Figure 3H shows that, averaged over a 5 s period, the shear stress at the bottom of the well did not exceed 0.02 Pa, well below the generally accepted upper limit of 1 Pa (10 dyne/cm^2^) for “low shear” conditions (Varma and Voldman, 2015). Based on these computational predictions, we expect to see increased nutrient availability for cells grown in these sub-wells without any detrimental effects from increased shear forces. These values are in agreement with low shear conditions found in rotating wall vessel bioreactors, used for successful tissue-specific differentiation of a variety of cells (Phelan et al., 2019; Begley and Kleis, 2000; Lelkes et al., 1998).

### 3.3 Electric current measurements and characterization

**FIGURE 4:**
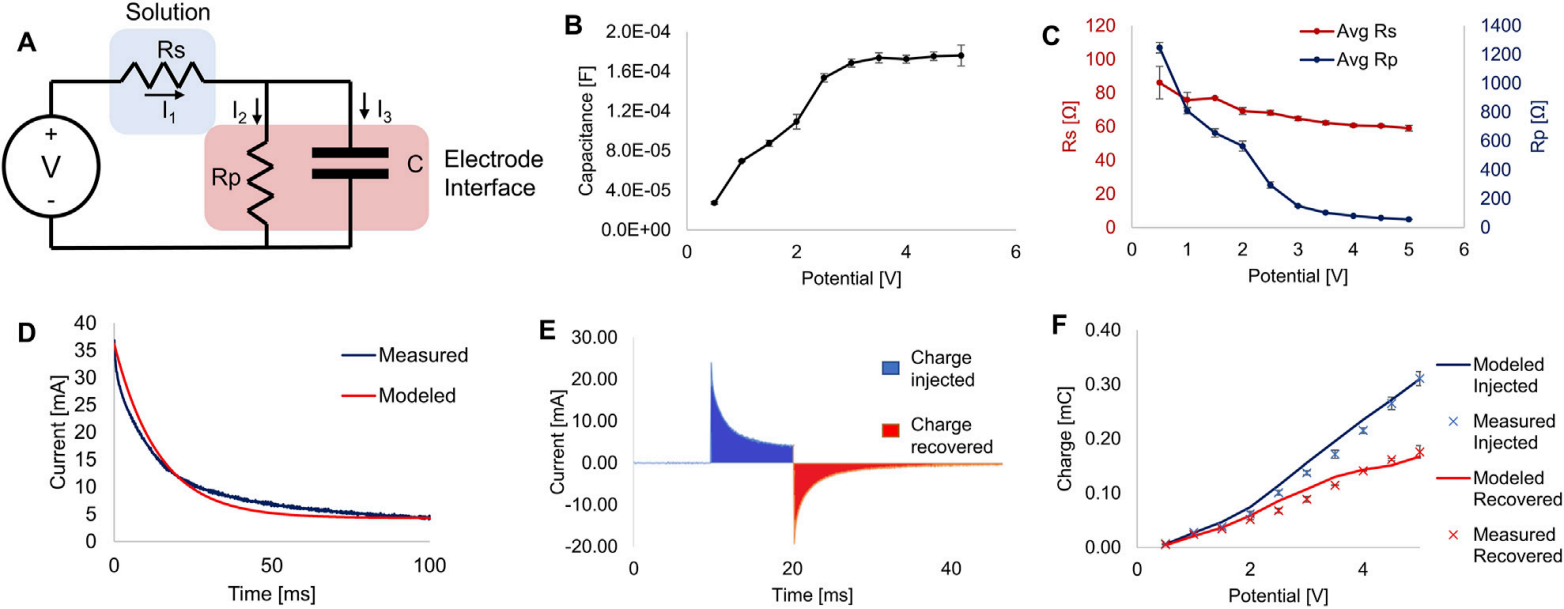
Electrical characterization. **(A)** Equivalent circuit of the bioreactor’s electrode-electrolyte system modeled as a simplified Randles circuit. **(B)** Estimated capacitance of the system calculated from current measurements at different applied potentials. **(C)** Estimates of the resistance calculated from current measurements at different applied potentials. **(D)** Example plot of measured current plotted against the modeled current of a single 100 ms pulse. **(E)** Example plot showing the injected and recovered charges from a 10 ms pulse. Filled sections show the areas integrated to calculate charge. **(F)** Plot of charge injected and recovered from 10 ms square pulses at various potentials, showing measured data and predictions made from mathematical modeling using estimated parameters. All error bars denote S.D., for n ≥ 3.

To fully understand the electrical signals acting on the cells in the bioreactor, we attempted to characterize and model the transient behavior of our electrical system. The movement of charge across an electrode-electrolyte interface led to a more complex system than the ideal charging of two parallel plates shown in the simplified computational modeling in Figures 3A–E. To more accurately characterize this system, we chose to model these interactions as a Randles circuit, (Figure 4A), as suggested in previous studies (Lu et al., 2013). In this model, Rs is the resistance of the electrolyte solution, C is the effective capacitance of the electrochemical double-layer, and Rp represents a polarization resistance, which effectively is the resistance to Faradaic charge transfer at this interface.

By measuring the current during a voltage step sufficiently long (500 ms) to reach steady-state current flow, we estimated the parameters for capacitance (Figure 4B), Rs and Rp (Figure 4C). We found this approach to be similar to previously reported methods and sufficient to determine the key parameters for our model system (Nelms et al., 2003). These values, especially C and Rp, changed significantly as the applied voltage changed. Plugging these parameters back into our model for each applied potential resulted in model-predicted current plots that closely matched our experimental recordings (see Figure 4D).

Having established a working mathematical model of the current through the cell culture media for any applied voltage, we then sought to quantify charge injection into the media at various applied potentials. Figure 4E shows an example recording of the current during and after a 10 ms monophasic square pulse, showing the areas integrated numerically over time to determine charge injected and charge recovered. Excess unrecovered charge is a sign of electrode degradation and potentially harmful byproduct formation, and thus should be minimized (Tandon et al., 2006). As seen in Figure 4F, model predictions of injected and recovered current closely matched the measured values. Thus, using this model, we will be able to accurately predict the current over time and charge injection for any applied voltage and pulse duration.

### 3.4 Optical analysis of cardiomyocytes

**FIGURE 5:**
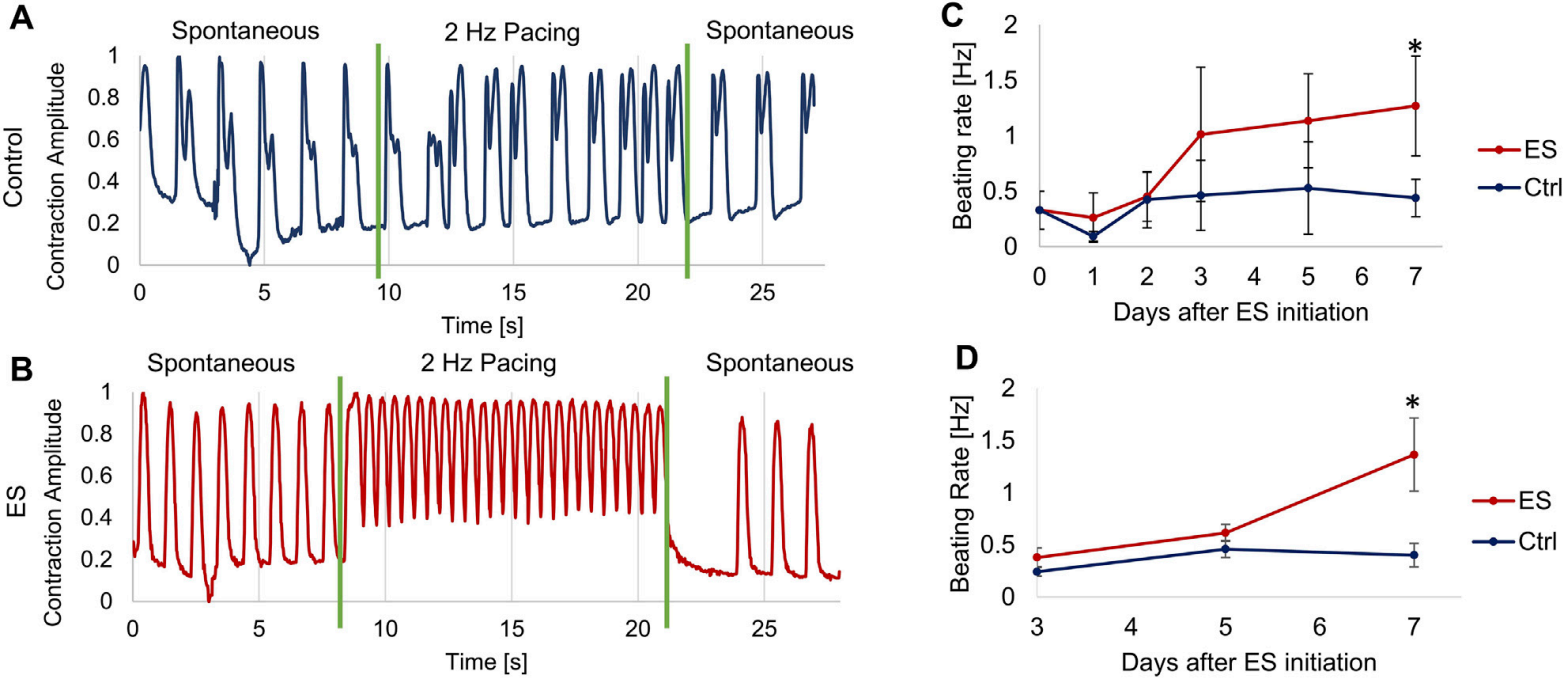
Measurement of cardiomyocyte contractions. **(A–B)** Optical measurements of CM beating before, during and after about 12 s of electrical pacing at 2 Hz, 5 V/cm, 10 ms pulse duration for control cells **(A)** and ES cells **(B)** on Day 5 after starting ES. During the 2 Hz pacing, the control cells **(A)** had a beating rate of 0.81 ± 0.169 Hz, and the ES cells **(B)** had a beating rate of 1.98 ± 0.11 Hz. **(C)** Spontaneous beating rate of monolayer CMs, average of at least 4 locations of 3 biological replicates per condition. **(D)** Spontaneous beating rate of cardiac spheroids, average of at least 5 spheroids per condition. Error bars indicate ±S.D., * indicates p < 0.05.

The beating rates of human induced pluripotent stem cell (hiPSC)-derived Cardiomyocytes (CMs) were assessed by analyzing phase-contrast video recordings of the beating CMs. Shown in Figures 5A, B are the responses of (A) control CMs or (B) CMs that had been exposed for 5 days to monophasic, pulsed electrical stimulation (ES) of 5 V/cm, 1Hz, 10 ms. Two days after the completion of this course of ES (7 days from the start) the spontaneous beating rate for the control CMs was 0.59 ± 0.014 Hz, compared to a beating rate of 0.95 ± 0.021 Hz for the ES CMs. After the onset of 2 Hz electrical pacing, the ES cells accurately captured the 2 Hz signal, beating at 1.98 ± 0.11 Hz, while the control cells increased their beating rate to only 0.81 ± 0.169 Hz. Thus, the CMs grown under control conditions, which had never before experienced external ES, were unable to fully capture the 2 Hz signal.

In a separate set of experiments, we determined the effect of long-term ES exposure on CM spontaneous beating rate. CMs were cultured either as monolayer or spheroid culture in the bioreactors and exposed to 5 V/cm, 1Hz, 10 ms monophasic pulsed ES for 5 days, followed by further 2 days without ES. Controls for both modes of culture were grown in the bioreactor, but without applied ES. Spontaneous beating rates were determined by first allowing the cells a “rest period” of at least 30 min without any ES before recording videos for analysis. For monolayer CMs (Figure 5C), the CMs exposed to ES increased to more than double the spontaneous beating rate of the control CMs on days 3–7, from 0.40 ± 0.21 to 1.27 ± 0.45 Hz, reaching significantly faster than the controls on day 7. The cardiac spheroids showed similar results (Figure 5D), with spheroids exposed to ES reaching an average beating rate of 1.36 ± 0.35 Hz on day 7 compared to 0.40 ± 0.11 Hz for the control group on the same day.

### 3.5 Maturation of cardiomyocytes

**FIGURE 6:**
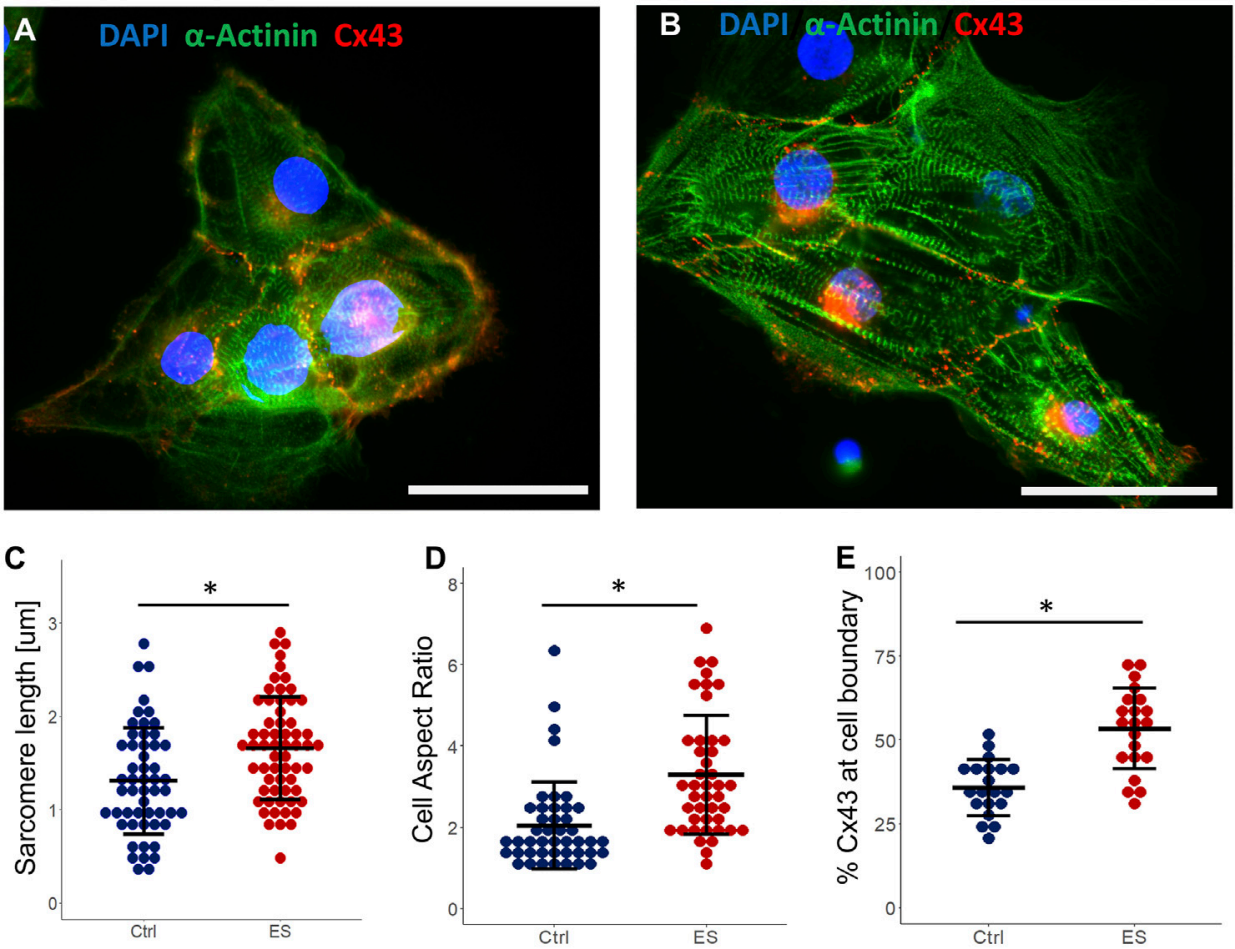
Cardiomyocyte maturation. **(A)** Control CMs stained for cardiac α-actinin, Connexin43, and DAPI. Scale bar = 25 μm. **(B)** ES CMs stained for cardiac α-actinin, Connexin43, and DAPI. Scale bar = 25 μm. **(C–E)** Quantification of sarcomere length **(C)**, cell aspect ratio (D) and Cx43 expression at cell boundary as a proportion of whole cell expression (E), from control and ES CMs. * indicates p < 0.05.

To examine how ES may have enhanced the structural maturation of hiPSC-CMs, we immunostained both control CMs and CMs exposed to ES for 5 days. Example images of cells stained for cardiac markers (cardiac alpha-actinin and Connexin43 (Cx43) are shown in Figures 6A, B. While staining of cardiac alpha-actinin showed organized sarcomeres in both groups, quantification of average sarcomere length for each group showed a significant increase (p < 0.05) from 1.31 ± 0.34 µm in the control group to 1.71 ± 0.29 µm for the ES group (Figure 6C). Though still lower than mature CMs, this increased sarcomere length indicates enhanced maturation for this population (Skorska et al., 2022a). Cell aspect ratio, another indicator of cardiomyocyte maturation (Ronaldson-Bouchard et al., 2018), was also significantly increased (p < 0.05) from 2.04 ± 1.07 in the control group to 3.34 ± 1.6 in the ES group (Figure 6D). To analyze Cx43 expression, we chose to quantify the localization of Cx43 to the cell periphery as a proportion of whole cell expression. Localization of Cx43 to cell junctions has been previously reported as a marker of CM maturation (Hamledari et al., 2022). Indeed, in line with our hypothesis, we found that ES of CMs in our bioreactor significantly increased (p < 0.05) the proportion of Cx43 localized to the cell periphery from 35.7% ± 8.4% in the control group to 53.4% ± 12.1% in the ES group (Figure 6E). Taken together, the data presented in panels 6C-6E suggest that the culture conditions in our bioreactor enhanced the structural maturation of hiPSC-derived CMs.

## 4 Discussion

In this study, we introduced a novel dynamic bioreactor designed to culture stem cell-derived cardiomyocytes concomitantly exposed to electrical stimulation and low-shear fluid flow. This bioreactor can be rapidly and inexpensively fabricated in-house through conventional 3D printing using biocompatible biomaterials (Figure 1). The unique design, with all cells being cultured directly on the bottom surface of standard 6-well tissue culture plates, allows for easy monitoring and imaging of cells during culture. Whether growing as 2D monolayers or 3D spheroids, there is no need to transfer cultures to another vessel for imaging or biochemical testing. The bioreactor system is compatible with any incubator or live cell imaging system that works with standard six-well plates. Because the 3D-printed plastic is press-fit to the bottom of the well, there is likely some leakage of fluids underneath the plastic sections. However, we have observed that cells cannot migrate underneath the plastic, meaning this gap is approximately 10 µm or less (Durante et al., 1993), resulting in slow enough leakage that this will not affect fluid dynamics. We also designed a custom signal generator (Figure 2) to provide the electrical signals for the bioreactors. Although previous custom, laboratory-made devices have been designed to provide similar signals in a biological setting (Moukarzel et al., 2021), for our purpose we desired a standalone device that could run reliably for days to weeks at a time.

We used computational tools to evaluate fundamental properties of fluid flow and the electric fields within the sub-wells. Modeling (Figure 3) showed that the electric field and current density were homogenous within the area of the sub-well in which the cells are cultured. This was important since it was essential to ensure that all cells would sense ES evenly throughout the well, to avoid any effects due to inhomogeneities in the electric current or voltage. Current density varied by less than 1% across the width of the sub-well, while the electric field was almost entirely homogenous. Computational fluid dynamic modeling confirmed that a stir bar spinning at up to 100RPM resulted in significant mixing in the center chamber, yet did not cause shear forces at the bottom of the sub-well to exceed 1Pa, which would be considered “low shear” for many cell types, such as fibroblast or endothelial cells (Varma and Voldman, 2015). A previous study focusing on the effects of shear stress on cardiomyocytes showed that while 1Pa of fluid shear would cause some changes to morphology and gene expression, no such changes were observed at 0.25Pa and below (Xu et al., 2022). Our maximum shear stress is estimated at about 0.025Pa, ten times lower than this previously reported threshold value. Thus, we posit that this mixing will be sufficient for increasing oxygen and nutrient availability to cells grown in the bioreactor without introducing any negative effects due to excess fluid shear. By simply changing the rotational speed of the stir bar we can change the amount of mixing, to enhance mass transport or introduce higher shear forces, if so desired.

Electrical stimulation has repeatedly been shown to aid in the *in vitro* differentiation and maturation of stem cell-derived cardiomyocytes. However, there is not yet a consensus on the best methods for providing that kind of stimulation (Dai et al., 2021). Researchers have used different bioreactor designs, with a wide variety of electrode materials and configurations, as well as varying patterns of stimulation signals. Nearly all published studies report that ES aids in cardiogenesis, but the degree of maturation and expression of markers for specific cardiac subtypes often disagree, even for studies which, on the surface, appear quite similar (Chan et al., 2013; LaBarge et al., 2019; Eng et al., 2016). We propose that these discrepancies may be due to a lack of proper characterization of the electrical signals used to stimulate the cells *in vitro*, which complicates comparing the effects of electrical stimulation between individual studies.

Prior studies have shown that different bioreactor designs and electrode materials contribute to variability in the response of cardiac cells to ES (Tandon et al., 2011). One of the most common ways of reporting the amplitude or “strength” of ES is to report the estimated electric field strength between the electrodes, often in units of V/cm. However, this number alone may not be enough to characterize the “strength” of an electrical signal, because the electric field is a function of both the applied potential and distance between the electrodes (Barash et al., 2010). Thus, with only the electric field reported, we cannot know the applied voltage without also knowing the geometry of the stimulation system. Importantly, without a defined signal and confirmation that the generated electric field is homogenous, we also cannot quantify the local current applied to the cells. Because cells in the myocardium are depolarized by local currents, it has been hypothesized that the level of electrical current, not the strength of the electric field, may be the most important factor in the stimulation of cardiomyocytes (Chen et al., 2009). The excitation threshold for cardiomyocyte stimulation *in vitro* reportedly changes based on the electrode material, which determines charge injection to the system (Tandon et al., 2011), further highlighting the importance and necessity of characterizing the electrical signals provided to cells in any bioreactor design.

Using simple current and voltage measurements, we characterized and modeled ES within the bioreactor. The electrode-electrolyte interface created by the interactions between parallel stainless-steel electrodes and the cell culture media was modeled using a simplified Randles circuit (Figure 4). Although the true interactions in this system were likely somewhat more complex than our simplified equivalent circuit (Merrill et al., 2005), this established model system suffices to characterize and predict current and charge injection in our bioreactor. The benefit of modeling the system of electrodes and fluid electrolyte as such a circuit is that it allows us to analytically solve for the current over time based on circuit parameters. Hence, we can estimate values for the parameters C, Rs, and Rp while knowing only the applied voltage and measured current through the entire system over time. Importantly, we can also use this model to calculate the charge injected into and recovered from the electrical system for any stimulation parameters that have been estimated (Lu et al., 2013). The ratio of charge injected to charge recovered can be important in a given biological system, as the non-recoverable charge is generally a result of faradaic reactions at the electrode interface (Wei and Grill, 2009). This leads to redox reactions in the electrolyte solution, resulting in the generation of potentially toxic compounds, such as reactive oxygen species (ROS).

Recoverable charge is primarily a result of non-faradaic charging of the electrochemical double-layer and is accounted for in our circuit model by current to the capacitive element. The current through the solution in this system varies non-linearly with applied voltage because the capacitance and polarization resistance vary based on the applied voltage (Shen et al., 2011). These same properties also mean that current varies significantly over time, even when considering the short, 2–50 ms pulse widths commonly used for ES of cardiac cells (Zimmermann et al., 2021). We hypothesize that full characterization of the electrical signals used will allow for better normalization and comparison between studies and enhance our knowledge of the effects of ES on cardiogenesis.

We tested our bioreactor by culturing hiPSC-derived CMs first as a monolayer and based our stimulation regimen on some common values used in previous studies: 5 V/cm electric field, 10 ms duration 1 Hz pulses (Eng et al., 2016), which in our bioreactor results in approximately 0.135 mC charge injected per pulse (Figure 4F). After 5 days of exposure to pulsed ES (with only brief breaks for measurements and media exchanges), the stimulated CMs had a faster spontaneous beating rate than control CMs (Figure 5), in line with previous studies on electrically stimulated cardiac cells (Bidez et al., 2011). The ES-conditioned cells also showed a much better response to external pacing. They fully captured a 2 Hz pacing rate, while the control CMs were unable to do so. To meet the eventual goal of transplantation into a damaged heart, hiPSC-CMs must be able to match the electrical and beating activity of native heart tissue (Inouye et al., 2024), which seems to be aided by pre-conditioning with ES.

Further evidence for enhanced maturation of CMs due to exposure to ES was seen when examining cells following staining for sarcomeric alpha-actinin and Connexin43 (Figure 6). The increased Cx43 expression on cell periphery that we observed may be a contributing factor to the faster beating and better pacing response seen in the ES-conditioned cells. Quantification of sarcomeres, visualized via actinin staining, showed significantly longer sarcomeres in the ES group compared to the control. During the immunostaining process, it is possible that fixation artifacts may have resulted in some slight shrinkage of the CM cell structures. However, given the consistency of the protocol used for all conditions, any comparisons made between the ES and control conditions should be valid. Thus, the longer sarcomeres seen in the ES group can be taken as a sign of CM maturity, which generally correlates to stronger cellular contractions (Skorska et al., 2022b). Similarly, another marker of CM maturation (Skorska et al., 2022b), the aspect ratio of cells (the ratio of the cell’s length to its width) is increased after exposure to ES. Taken together, these results show that ES leads to an increase in the structural organization and maturity of hiPSC-derived cardiomyocytes.

The results of this work may be of significant benefit for future studies requiring scale-up of a bioreactor system capable of electrical stimulation and low-shear fluid flow. This is particularly true for scale-up in terms of higher throughput screening applications (Wei et al., 2020), or to meet the need for larger numbers of cells or tissues needed for therapeutic purposes (Sougawa et al., 2021), both of which are likely uses for hiPSC-derived CMs.

## 5 Conclusion

In this study we presented a novel bioreactor system capable of electrical stimulation while enhancing mass transport (supply of oxygen and nutrients) of cultured hiPSC-derived cardiomyocytes. We demonstrated a method of growing cells exposed to ES while allowing continuous, easy observation, due to the bioreactor’s “drop-in” design. Our bioreactor also provided a gentle, continuous fluid flow for enhanced mixing and nutrient availability. Importantly, the ES produced by this system was fully characterized and modeled, allowing for exact knowledge and reproducibility of stimulation parameters, including voltage, current, and injected/recovered charge. The results presented here are based on well-characterized computational/electrical stimulation parameters and thus should be easily repeatable by future studies. Understanding of and control over critical parameters, such as current and charge injection, will yield data that are comparable across systems and investigators. Matching only a single parameter, such as stimulation frequency or voltage, is not enough to ensure similar growth conditions within two different bioreactor setups. With this dynamic bioreactor for electrical stimulation, we hope to advance the field of cardiomyocyte differentiation by increasing the comparability and repeatability of *in vitro* studies such as these. We are confident that future studies using our bioreactor platform will help to elucidate exactly which ES parameters are critical for altering the maturation and subtype specification of hiPSC-CMs.

## Data Availability

The original contributions presented in the study are included in the article/supplementary material, further inquiries can be directed to the corresponding author.
